# Immunogenicity of Bivalent Human Papillomavirus DNA Vaccine Using Human Endogenous Retrovirus Envelope-Coated Baculoviral Vectors in Mice and Pigs

**DOI:** 10.1371/journal.pone.0050296

**Published:** 2012-11-27

**Authors:** Hee-Jung Lee, Yoon-Ki Hur, Youn-Dong Cho, Mi-Gyeong Kim, Hoon-Taek Lee, Yu-Kyoung Oh, Young Bong Kim

**Affiliations:** 1 College of Animal Bioscience & Technology, Konkuk University, Seoul, South Korea; 2 College of Pharmacy and Research Institute of Pharmaceutical Sciences, Seoul National University, Seoul, South Korea; UC Irvine Medical Center, United States of America

## Abstract

Human papillomavirus is known to be the major pathogen of cervical cancer. Here, we report the efficacy of a bivalent human papillomavirus type 16 and 18 DNA vaccine system following repeated dosing in mice and pigs using a recombinant baculovirus bearing human endogenous retrovirus envelope protein (AcHERV) as a vector. The intramuscular administration of AcHERV-based HPV16L1 and HPV18L1 DNA vaccines induced antigen-specific serum IgG, vaginal IgA, and neutralizing antibodies to levels comparable to those achieved using the commercially marketed vaccine Cervarix. Similar to Cervarix, AcHERV-based bivalent vaccinations completely blocked subsequent vaginal challenge with HPV type-specific pseudovirions. However, AcHERV-based bivalent vaccinations induced significantly higher cell-mediated immune responses than Cervarix, promoting 4.5- (HPV16L1) and 3.9-(HPV18L1) fold higher interferon-γ production in splenocytes upon stimulation with antigen type-specific pseudovirions. Repeated dosing did not affect the immunogenicity of AcHERV DNA vaccines. Three sequential immunizations with AcHERV-HP18L1 DNA vaccine followed by three repeated dosing with AcHERV-HP16L1 over 11 weeks induced an initial production of anti-HPV18L1 antibody followed by subsequent induction of anti-HPV16L1 antibody. Finally, AcHERV-based bivalent DNA vaccination induced antigen-specific serum IgG immune responses in pigs. These results support the further development of AcHERV as a bivalent human papillomavirus DNA vaccine system for use in preventing the viral infection as well as treating the infected women by inducing both humoral and cell-mediated immune responses. Moreover, the possibility of repeated dosing indicates the utility of AcHERV system for reusable vectors of other viral pathogen vaccines.

## Introduction

DNA vaccines have been studied as next-generation vaccines that may replace current subunit or live-attenuated vaccines. DNA vaccines offer several advantages compared to conventional vaccines, including relative stability and safety, capacity to induce cell-mediated immune responses, and ease of manipulation. Moreover, they can be created using less complex production process and are thus less expensive to produce on a large scale. Despite these advantages and initial high hopes, research progress in this area since the first report about two decades ago has been slow, with only a few DNA vaccines reaching clinical trials to date [1], [2]. One major limitation that has hampered the successful development of DNA vaccines is the intracellular delivery issue. Because of their highly negative charge and large size, naked plasmid DNA cannot effectively penetrate the cell membrane [3], [4].

To improve the efficacy of DNA vaccine cellular delivery, researchers have investigated various nonviral and viral vectors. Nonviral cationic liposomes [5] and polymers [6] have been studied as delivery systems for plasmid DNA vaccines, and physical (electroporation) methods have been applied for introducing human papillomavirus (HPV) DNA into cells [7], [8]. Among the viral vectors investigated as delivery systems for antigen-encoding DNA are recombinant adenovirus [9] and vaccinia virus [10]. Although viral vectors have advantages over nonviral vector systems in terms of intracellular delivery efficacy, they suffer from at least two major drawbacks from the standpoint of clinical development. First, most viral vectors can be converted to pathogenic forms after replications. Second, viral vectors are immunogenic, limiting repeated dosing with DNA vaccines.

Overcoming the limitations of currently studied viral vectors requires the development of new viral vectors that are non-replicating in human cells (eliminating the potential conversion to pathogenic forms) and non-immunogenic (allowing repeated dosing with DNA vaccines) [11]. Previously, we reported the use of non-replicating recombinant baculoviral vectors as an HPV16 DNA vaccine nanocarrier system [12]. Baculoviruses replicate in insect cells, but not in human cells; however, they cannot effectively enter human cells. To facilitate the intracellular delivery function, we engineered the baculovirus to express the human endogenous retrovirus (HERV) envelope gene (*env*). HERV envelope protein-coated, non-replicating baculoviral vectors (AcHERV) showed increased delivery into human and murine cells compared to baculoviral vectors without HERV *env* [12]. However, this previous study [12] left several questions regarding in vivo function unanswered.

In this study, we first tested whether the AcHERV system is capable of functioning as a delivery system for bivalent HPV16L1 and HPV18L1 DNA vaccines. Given the multiple types of viral infections involved in the pathogenicity of infectious diseases [13], [14], demonstrating the feasibility of bivalent DNA vaccination may provide insight into the future application of this system for multivalent DNA vaccine delivery. Secondly, we tested whether the AcHERV vectors retained their DNA vaccine-delivery efficacy in vivo after multiple dosing. Finally, we tested the immunogenicity of AcHERV-based bivalent DNA vaccines in mice and pigs. We report that the AcHERV-based bivalent DNA vaccines tested here induced humoral immune responses sufficient to block subsequent infection by pseudoviruses, and led to significantly elevated cell-mediated immune responses. Moreover, multiple in vivo administrations of the AcHERV-based DNA vaccines did not reduce immunogenicity, and AcHERV-based bivalent DNA vaccines induced immune responses in both mice and pigs.

## Materials and Methods

### Ethics Statement

The use of animals in this experiment was approved by Institutional Animal Care and Use Committee (IACUC) of Seoul National University (Approval No. SNU-201003-02). All animal experiments were conducted in accordance with the animal care guideline of Seoul National University, Korea.

### Construction of Recombinant Baculoviruses

**Figure 1:**
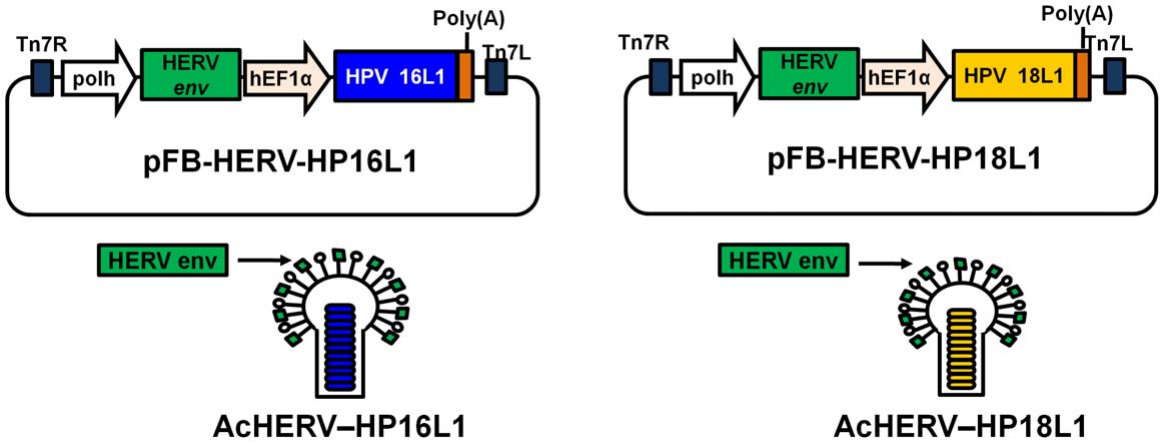
Schematic diagrams of recombinant baculoviruses expressing HPV16L1 or HPV18L1. A pFastBac1 plasmid was constructed to contain the hEF1α promoter and the polyhedrin promoter controlling HERV *env* expression, pFB-HERV-HP16L1, or pFB-HERV-HP18L1. Recombinant baculoviruses AcHERV-HP16L1 and AcHERV-HP18L1 were generated using the Bac-to-Bac baculovirus expression system, and AcHERV-HP16L1 or AcHERV-HP18L1.

A recombinant baculovirus vector expressing HERV *env* (pFastBac-HERV) was constructed by inserting a synthetic codon-optimized envelope gene of HERV type W (GenBank accession number NM014590, GenScript Corp., Piscataway, NJ, USA) into pFastBac1 (Invitrogen, Carlsbad, CA, USA). Next, pFastBac-HERVs encoding HPV16 L1 (pFB-HERV-HP16L1), HPV18L1 (pFB-HERV-HP18L1), or enhanced green fluorescent protein (pFB-HERV-eGFP) were constructed by inserting each gene into *Nco*I and *Nhe*I sites of pFastBac-HERV [12]. HERV envelope protein-expressing recombinant baculoviruses (AcHERV) encoding HPV16L1 (AcHERV-HP16L1), 18L1 (AcHERV-HP18L1), or enhanced green fluorescent protein (AcHERV-eGFP) were produced using the Bac-to-Bac Baculovirus Expression System (Invitrogen), according to the manufacturer’s instructions. The construction scheme for recombinant baculoviruses is shown in Figure 1.

### Generation of HPV16 and HPV18 Pseudoviruses

HPV16 and HPV18 pseudoviruses were prepared as described previously [15] by co-transfection of 293TT cells with p16L1/L2 or p18L1/L2 plasmids, together with pSEAP (secreted alkaline phosphatase) or pLucf (luciferase) marker plasmid. After incubation at 37°C for 48 hours, cells were lysed by adding Triton X-100 (Sigma, St. Louis, MO, USA) at a final concentration of 0.5% in Dulbecco’s phosphate-buffered saline (DPBS) supplemented with 9.5 mM MgCl_2_. Lysates were digested for 24 hours at 37°C with 0.2% Benzonase (Sigma) to complete virus maturation. The lysate was mixed with 0.8 M NaCl and clarified by centrifugation at 2,000×g for 15 minutes. Pseudoviruses were purified on an OptiPrep step gradient by centrifugation at 234,000×g for 4 hours. After centrifugation, fractions were collected and stored at -80°C [16].

### Western Blot Analysis

Western blotting was used to test the expression of HPV16L1 and HPV18L1 after cellular delivery using AcHERV vectors. 293TT cells (ATCC, Rockville, MD, USA) cultured in Dulbecco’s Modified Eagle Medium (DMEM) containing 10% fetal bovine serum (FBS) were treated with HPV16L1 pseudoviruses, HPV18L1 pseudoviruses, or purified recombinant baculoviruses (AcHERV-HP16L1 or AcHERV-HP18L1) at a multiplicity of infection (MOI) of 10. Three days after infection, 293TT cells were lysed by incubating with lysis buffer. Lysates of 293TT cells were resolved by sodium dodecyl sulfate-polyacrylamide gel electrophoresis on 10% gels and transferred to nitrocellulose membranes. HPV L1 protein was detected by first incubating the membranes with primary anti-HPV L1 antibodies (obtained from the serum of mice immunized with AcHERV-HP16L1 or AcHERV-HP18L1). As a protein loading control, ß-actin antibody (1∶2,000 dilution, Santa Cruz Biotechnology, Santa Cruz, CA, USA) was used Secondary antibody was alkaline phosphatase-conjugated monoclonal goat anti-mouse secondary antibody (1∶3,000 dilution; Santa Cruz Biotechnology).

### Co-immunization of Mice with AcHERV-HP16L1 and AcHERV-HP18L1

Four-week-old female BALB/c mice (Orient-Bio, Kyonggi-do, Korea) were simultaneously or sequentially co-immunized with AcHERV-HP16L1 and AcHERV-HP18L1. For simultaneous co-immunization, a mixture of AcHERV-HP16L1 and AcHERV-HP18L1 (AcHERV-HP16/18L1) was administered to mice as a single injection dose of 1×10^7^ PFU for each recombinant baculovirus. Mice were treated with AcHERV-HP16/18L1 three times at 2-week intervals. For sequential co-immunization, mice were first immunized three times with AcHERV-HP18L1 (1×10^7^ PFU/dose) at 2-week intervals, and then three times with AcHERV-HP16L1 (1×10^7^ PFU/dose) at 2-week intervals. For comparison, mice were immunized three times with Cervarix (GlaxoSmithKline, Middlesex, UK) at a dose of 0.1 mg/kg (1/20^th^ of a human dose). Sera and vaginal washes from each group were obtained 0, 1, 3, 5, 10, and 20 weeks after the first immunization.

### Co-immunization of Pigs with AcHERV-HP16L1 and AcHERV-HP18L1

Miniature pigs, kindly donated by Dr. Yoon Berm Kim (Rosalind Franklin University of Medicine and Science, CMS, Chicago, IL, USA), were maintained under specific pathogen-free condition. Two-year-old pigs weighing ∼130 kg were simultaneously co-immunized with AcHERV-HP16L1 and AcHERV-HP18L1 at a dose of 3×10^8^ PFU each, intramuscularly injected behind the ear, three times at 3-week intervals. Three, six and nine weeks after the first injection, serum samples were collected for serological tests.

### Enzyme-linked Immunosorbent Assay (ELISA)

HPV16- and 18L1-specific antibody production was tested by ELISA using pseudoviruses as coating antigens. Sixty microliters of pseudovirus (0.001 mg/ml) was added to each well of a 96-well plate and incubated for 16 hours at 4°C. Plates were then washed and blocked with 2% (w/v) bovine serum albumin in PBS containing 0.1% Nonidet P-40 (Sigma). Serially diluted mouse sera or vaginal washings (0.06 ml/well) were added and incubated at room temperature for 2 hours. After washing, peroxidase-conjugated goat anti-mouse IgG antibody (1∶2000; Santa Cruz Biotechnology) or goat anti-mouse IgA antibody (1∶1000; Santa Cruz Biotechnology) was added. For color development, 1-Step Turbo TMB (3,3′,5,5′-tetramethyl benzidine substrate solution; Pierce, Rockford, IL, USA) was added. Endpoint titers were defined as the highest serum dilutions that resulted in an absorbance value twice that of non-immunized serum (cutoff value, 0.1) and were expressed as the group geometric means ± SDs.

### Neutralization Assay

Neutralizations assays were performed using SEAP-expressing HPV16 pseudoviruses according to a previously described method [17]. Briefly, OptiPrep-purified SEAP HPV16 pseudoviruses were diluted 3,000-fold and incubated on ice for 1 hour with 3-fold serial dilutions of vaginal washes. 293TT cells were infected by incubating with pseudovirus–antibody mixtures for 3 days. The SEAP content in 10 µl of clarified cell supernatant was determined using a Great EscAPe SEAP Chemiluminescence Kit (Clontech, Mountain View, CA, USA). Neutralization titers were defined as the reciprocal of the highest serum dilution that caused at least a 50% reduction in SEAP activity.

### Challenge Test with HPV Pseudoviruses

Twenty weeks after the first co-immunization with AcHERV-HP16L1 and AcHERV-HP18L1, mice were challenged with HPV pseudoviruses, as described previously [18], [19]. Eight days before in vivo genital challenge with pseudoviruses, mice were synchronized in a diestrus-like status by subcutaneous injection with 3 mg DepoProvera (Pfizer AG, Zurich, Switzerland). Six hours prior to pseudovirus challenge, deeply anesthetized mice were pretreated intravaginally with 20 µl of 4% nonoxynol-9 (Sigma). Mice were genitally challenged with 5×10^6^ IU of HPV17 and HPV18 pseudoviruses, each in a 20-µl solution containing 2% carboxymethylcellulose (Sigma). Three days later, all mice were anesthetized and injected intraperitoneally with luciferin (30 µl at 7 mg/ml) to detect luciferase expressed upon pseudoinfection by pseudoviruses, which encapsidate pLucf, a plasmid carrying a luciferase gene (http://home.ccr.cancer.gov/lco/). The expression of luciferase was detected by measuring light emission over 10 minutes with an IVIS 200 bioluminescence imaging system (Xenogen, Cranbury, NJ, USA Equal-sized areas encompassing the site of virus inoculation were analyzed using Living Image 2.20 software (Xenogen).

### Enzyme-linked Immunospot (ELISPOT) Assay

The production of gamma interferon (IFN-*γ*) from splenocytes of immunized mice was detected by ELISPOT assay. A 96-well plate was coated with 0.2 µg of anti-mouse IFN-*γ* capture antibody, and then blocked by incubating with 10% FBS at 37°C. Splenocytes were seeded at 1×10^6^ cells per well in 100 µl of medium, and stimulated by adding 1×10^6^ HPV pseudoviruses and incubating for an additional 24 hours at 37°C. Plates were then washed with PBS containing 0.05% Tween-20 and treated with 20 ng of biotinylated anti-mouse IFN-*γ* detection antibody. After 2 hours, streptavidin-alkaline phosphatase was added to the wells, and color was developed with an AEC substrate reagent (BD Biosciences, Franklin Lakes, NJ, USA). The number of spots was counted using an ELISPOT reader (AID ElispotReader ver.4; Strassberg, Germany).

### Statistical Analysis

All data were analyzed by ANOVA with Student-Newman-Keuls as a post hoc test using SigmaStat software (Systat Software, Richmond, CA, USA). P-values less than 0.05 were considered significant.

## Results

### Construction of AcHERV-HP16L1 and AcHERV-HP18 L1

**Figure 2:**
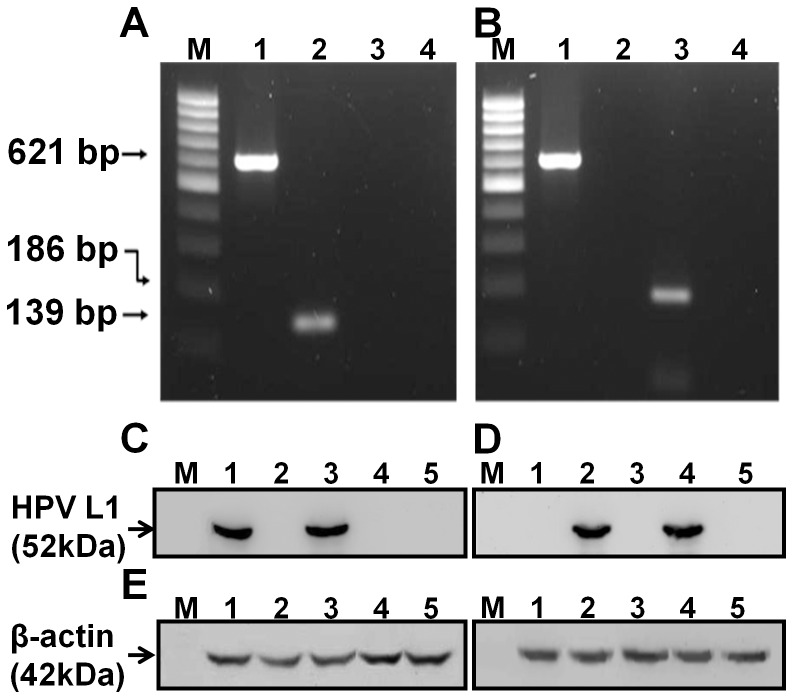
Characterization of recombinant baculovirus and expression of HPV16 or HPV18 L1. AcHERV-HP16L1 (A) and AcHERV-HP18L1 (B) were generated by amplification of HERV *env* and HPV L1 genes from viral DNA using specific PCR primers. Controls were prepared by omitting templates from the sample. M: 100-bp ladder; Lane 1: HERV env; Lane 2: HPV 16L1; Lane 3: HPV 18L1; Lane 4: Control. Western blotting was performed on extracts of HPV16 pseudovirus-, HPV18 pseudovirus-, AcHERV-HP16L1- or AcHERV-HP18L1-infected 293TT cells using anti-HPV16L1 (C), anti-HPV18L1 (D), and anti- ß-actin antibodies (E). M: protein marker; Lane 1: lysate from cells infected with HPV16 pseudovirus; Lane 2: lysate from cells infected with HPV18 pseudovirus; Lane 3: lysate from cells infected with AcHERV-HP16L1; Lane 4: lysate from cells infected with AcHERV-HP18L1; Lane 5: lysate from uninfected cells.

We previously reported the construction of AcHERV vector for HPV16L1 (AcHERV-HP16L1). Here, to test the feasibility of AcHERV vectors as multivalent DNA vaccine delivery systems, we constructed an AcHERV vector for HPV18L1 (AcHERV-HP18L1) using the same HERV *env* and human elongation factor 1-α promoter system (Fig. 1). PCR confirmed the presence of HERV *env* in both AcHERV-HP16L1 (Fig. 2A, lane 1) and AcHERV-HP18L1 (Fig. 2B, lane 1), and the unique presence of the HPV16 L1 gene in AcHERV-HP16L1 (Fig. 2A, lane 2) and the HPV18L1 gene in AcHERV-HP18 L1 (Fig. 2B, lane 3). Western blot analyses showed that cells treated with either AcHERV-HP16L1 (Fig. 2C, lane 3) or AcHERV-HP18L1 (Fig. 2D, lane 4) expressed HPV L1 proteins with an approximate molecular weight of 52 kD. Moreover, pseudoviral HPV L1 was detected by antibodies in a type-specific manner. Anti-HPV16 L1 antibodies did not cross react with L1 from HPV18 pseudovirus (Fig. 2C, lane 2) or AcHERV-HP18L1-treated groups (Fig. 2C, lane 4). Similarly, anti-HPV18 L1 antibodies did not cross react with L1 from HPV16 pseudovirus (Fig. 2D, lane 1) or AcHERV-HP16L1-treated groups (Fig. 2D, lane 3).

### Humoral Immune Responses Following Bivalent Immunization in Mice

**Figure 3:**
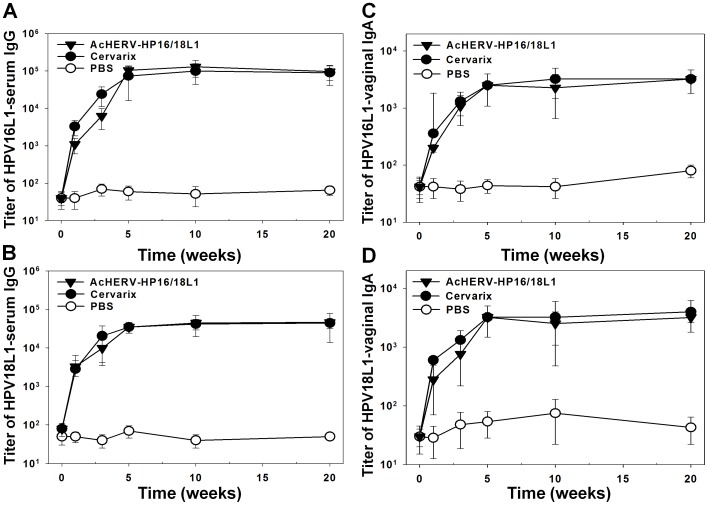
Titers of antibodies specific for HPV16L1 and HPV18L1 in mouse sera. BALB/c mice were intramuscularly immunized with Cervarix, PBS (control), or 1×10^7^ PFU of recombinant AcHERV-HP16/18L1. Antigen-specific IgG antibody titers against HPV16L1 (A) or HPV18L1 (B) in murine vaginal washes were determined by ELISA. Antigen-specific IgA antibody titers against HPV16L1 (C) or HPV18L1 (D) in murine vaginal washes were determined by ELISA.

Because HPV types 16 and 18 are responsible for 70% of cervical cancers and 85% of anal cancers [15], [20], and for purposes of comparison with the HPV vaccine Cervarix, which is composed of HPV16 and 18 bivalent virus-like particles (VLPs), we co-immunized mice with AcHERV-HP16L1 and AcHERV-HP18L1 as a bivalent DNA vaccine. After co-immunization, HPV L1 type-specific serum IgG and vaginal IgA were induced to levels comparable to those in the group treated with Cervarix (Fig. 3). From 1 week after the first immunization, HPV 16 and 18 L1-specific IgG were detected in mice co-immunized with AcHERV-HP16L1 and AcHERV-HP18L1. At 5 weeks after the first administration, the induction of serum IgG antibodies specific for HPV 16 (Fig. 3A) and 18 L1 (Fig. 3B) reached a plateau level that was subsequently maintained for at least 15 more weeks (20 weeks after the first administration). Neither anti-HPV16L1- nor HPV18L1-specific IgG levels following co-immunization with AcHERV-HP16L1 and AcHERB-HP18L1 differed significantly from those induced by Cervarix.

Because mucosal antibodies are crucial in protecting against sexually transmitted infections and form the first line of defense against such infectious agents [21], the secretory IgA response is an important marker of mucosal immunity. Following co-administration of AcHERV-HP16L1 and AcHERV-HP18L1, vaginal IgAs specific for HPV16L1 (Fig. 3C) or HPV18L1 (Fig. 3D) were induced with kinetics similar to those of serum IgG, reaching a plateau at 5 weeks after the first administration and persisting for at least 20 weeks. Moreover, there was no significant difference in the levels of vaginal IgA antibody induced by co-immunization and Cervarix treatment.

### Induction of Neutralizing Antibody after Bivalent Immunization in Mice

**Figure 4:**
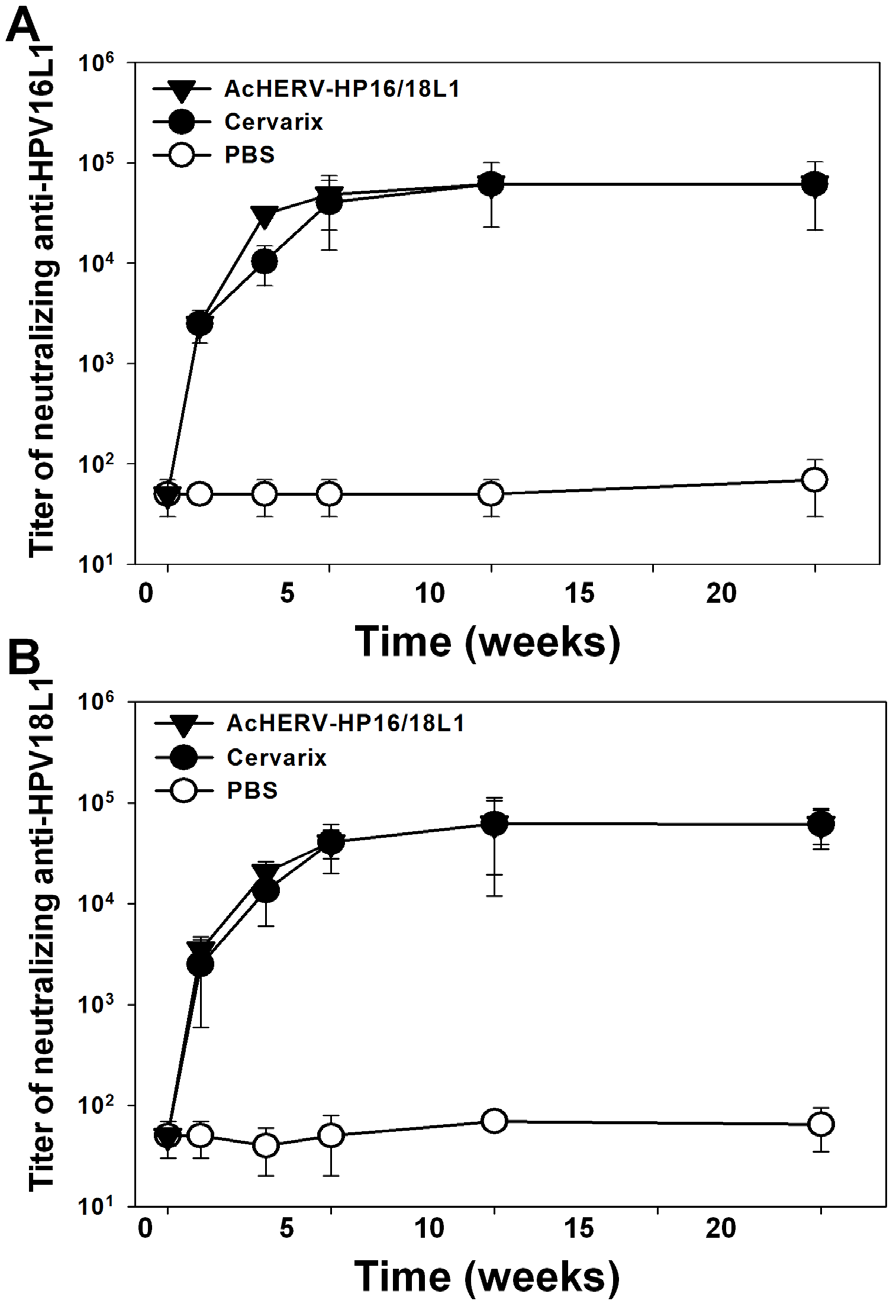
Induction of HPV16L1- or HPV18L1-specific neutralizing antibodies in mice after immunization with recombinant baculoviruses. Serum was sampled at 1 to 20 weeks after immunization with recombinant baculoviruses, Cervarix, or PBS. Neutralization assays were performed with HPV16 (A) or HPV18 (B) pseudoviruses. Data are expressed as the geometric means (log) of reciprocal serum dilutions that yielded a 50% reduction in SEAP.

In addition to inducing serum IgG and vaginal IgA immune responses, bivalent immunization with AcHERV-HP16L1 and AcHERV-HP18L1 substantially induced neutralizing antibody in mice. After a total of three immunizations, both HPV16L1-specific (Fig. 4A) and HPV18L1-specific (Fig. 4B) neutralizing antibodies were induced at a maximal level, and persistent until 20 weeks after the first administration. Consistent with HPVL1-specific serum IgG induction data, neutralizing antibody-induction capability and kinetics did not differ between the group administered bivalent AcHERV vaccine and the Cervarix-treated group.

### Challenge with HPV Pseudoviruses in Mice

**Figure 5:**
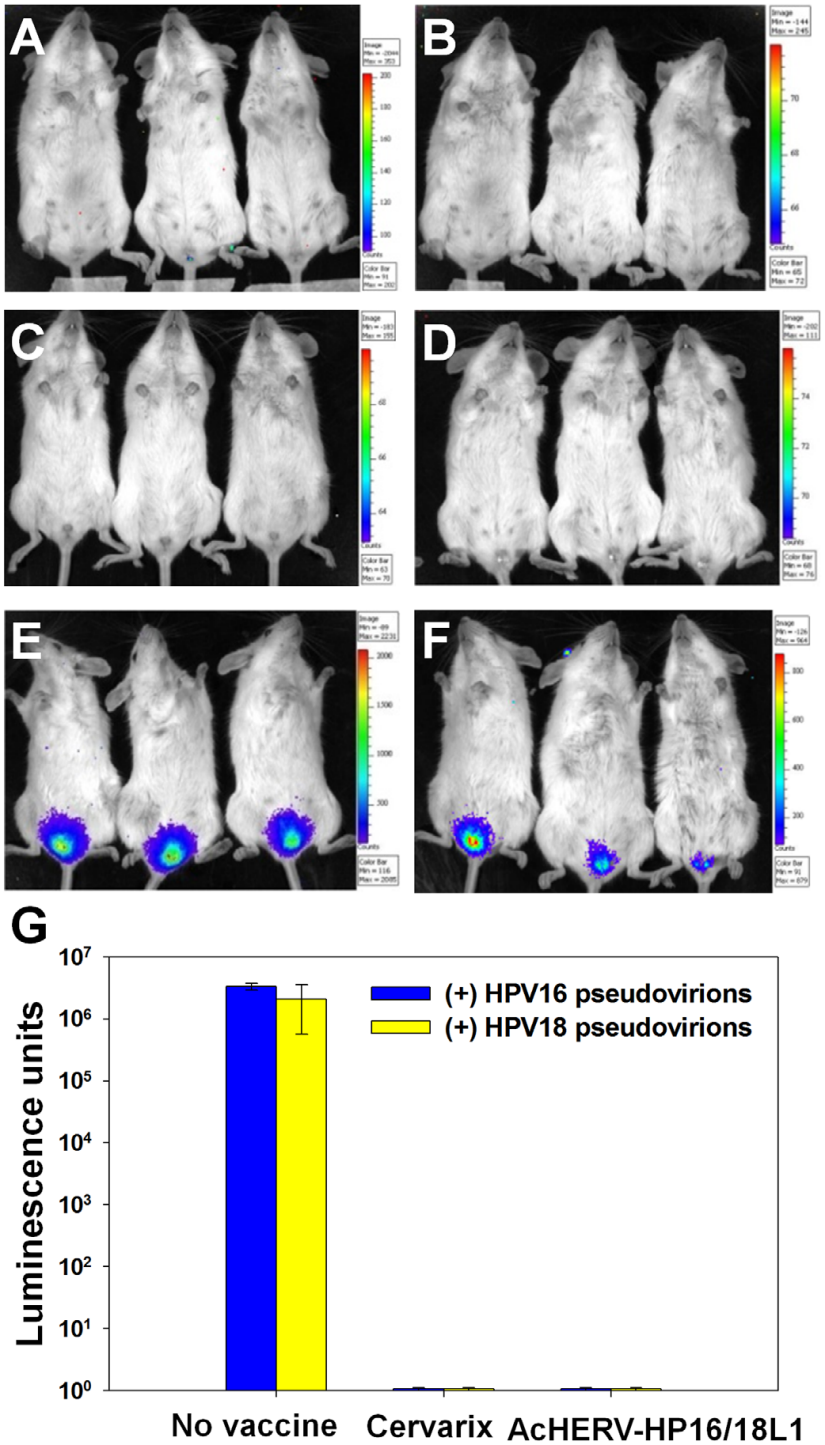
HPV challenge test with HPV16 or HPV18 pseudovirions in AcHERV-HP16/18L1- or Cervarix-vaccinated mice. Mice immunized with AcHERV-HP16/18L1 (A and B), Cervarix (C and D), or PBS (E and F) received a vaginal challenge with HPV16 (A, C, and E) or HPV18 (B, D, and F) infectious pseudoviruses at 20 weeks after the first immunization. Three days after challenge, mice were anesthetized, injected with luciferin, and imaged for 10 minutes with a Xenogen IVIS 200 bioluminescence imaging system. (G) Equal-sized areas encompassing the site of inoculation were analyzed using Living Image 2.20 software and plotted after background subtraction.

To test whether neutralizing antibody titers generated by co-immunization with AcHERV-HP16L1 and AcHERV-HP18L1 were sufficient to mediate protection in mice, we challenged bivalent DNA-vaccinated mice with HPV16 or HPV18 pseudovirus via the vaginal route. Vaginal pseudoinfection with HPV16 or HPV18 pseudovirus was detected by monitoring the expression of the luciferase reporter gene using whole-organ, multispectral molecular imaging [18]. Non-immunized mice challenged with HPV16 (Fig. 5E) or HPV18 (Fig. 5F) pseudovirus exhibited luciferase expression, reflecting effective vaginal pseudoinfection by pseudoviruses carrying the luciferase gene. In contrast, immunization of mice with bivalent AcHERV-HPV16L1 and AcHERV-HP18L1 provided almost complete protection against pseudoinfection by HPV16 (Fig. 5A) and HPV18 (Fig. 5B) pseudoviruses. Similarly, mice immunized with Cervarix showed no detectable levels of luciferase expression following challenge with HPV16 (Fig. 5C) or HPV18 (Fig. 5D) pseudovirus. Quantitative analyses of the images showed that immunization with the AcHERV bivalent DNA vaccine reduced luciferase expression by more than six orders of magnitude compared to the non-immunized group.

### IFN-γ Immune Response in Mice

**Figure 6:**
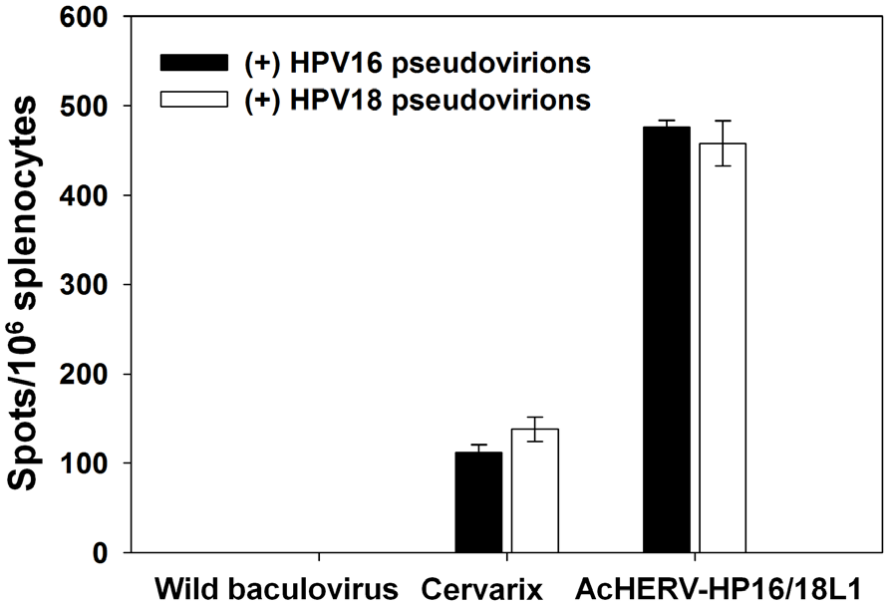
ELISPOT assay of IFN-γ-producing HPV16- or HPV18-specific T cells. Mouse splenocytes were harvested 20 weeks after the first immunization. The number of IFN-γ-producing HPV16- or HPV18-specific T cells was determined using an ELISPOT assay. Values represent the number of spots per 10^6^ splenocytes following stimulation with HPV16 or HPV18 pseudoviruses.

In addition to humoral immune responses, cell-mediated immune responses were induced by immunization with bivalent AcHERV HPV vaccines (Fig. 6). The elicitation of cellular immunity was determined by measuring IFN-**γ** production from splenocytes stimulated with HPV 16 or 18 pseudovirus. Intramuscular injection of mice with wild-type baculovirus lacking HERV *env* and HPVL1 genes did not induce the production of IFN-**γ** by splenocytes. Although treatment with Cervarix induced detectable levels of IFN-**γ** production by splenocytes upon stimulation with HPV16 or 18 pseudovirus, IFN-**γ** production was significantly higher in AcHERV bivalently vaccinated mice. In these mice, stimulation with HPV16 and HPV18 pseudovirus induced increases in IFN-**γ** production that were 4.5- and 3.9-fold greater, respectively, than those induced by Cervarix.

### Serum Antibody Responses following Multiple Immunizations in Mice

**Figure 7:**
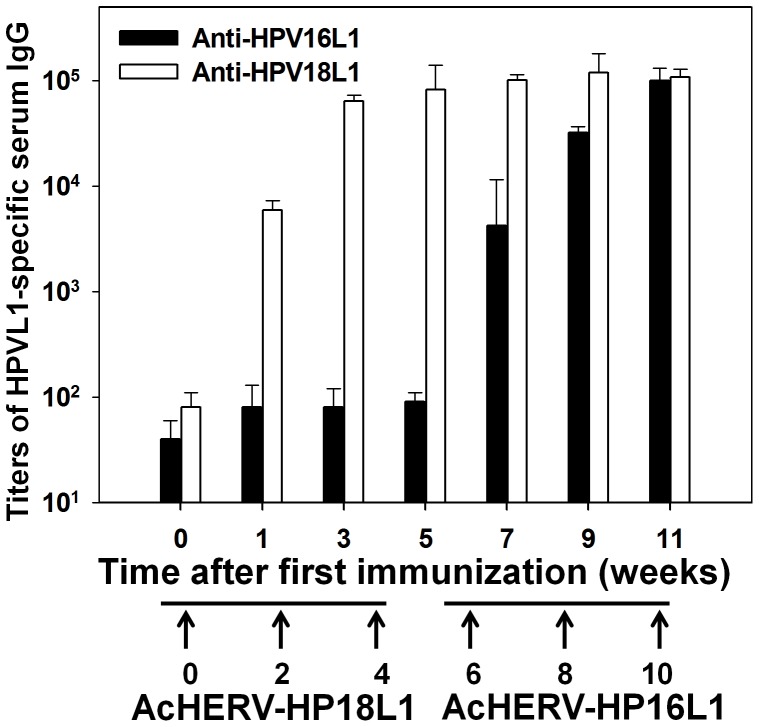
Sequential immunization with AcHERV-HP18L1 and AcHERV-HP16L1. BALB/c mice were intramuscularly immunized with 1×10^7^ PFU of AcHERV-HP18L1 three times at 2-week intervals and then with 1×10^7^ PFU of AcHERV-HP18L1. Antigen-specific antibody IgG titers were determined by ELISA.

A multiple dosing schedule is not possible with most viral vectors owing to the development of antibodies against viral vectors, a serious limitation to the application of DNA vaccination strategies. Accordingly, we next tested whether a multiple immunization protocol is possible with AcHERV vectors. To answer this question, we administered repeated immunizations, delivering the first three immunization at 0, 2, and 4 weeks using AcHERV-HP18L1, and the next three immunizations at 6, 8, and 10 week using AcHERV-HP16L1. The injection frequency is indicated by arrows in Figure 7. Following the first three immunizations with AcHERV-HP18L1, anti-HPV16 antibody was detected at background levels in serum through 5 weeks after the first immunization, but anti-HPV18 antibody levels in serum continued increasing following booster doses, reaching peak levels within 3 weeks after the first immunization (Fig. 7). Subsequent immunizations with AcHERV-HP16L1 were capable of inducing type-specific anti-HPV16 L1 antibodies, yielding a significant increase in anti-HPV16 L1 antibody 1 week after the first AcHERV-HP16L1 administration. Five week after the first AcHERV-HP16L1 administration, anti-HPV16 L1 antibody titers were similar to anti-HPV18L1 antibody titers.

### Serum Antibody Responses Following Bivalent Immunization in Pigs

**Figure 8:**
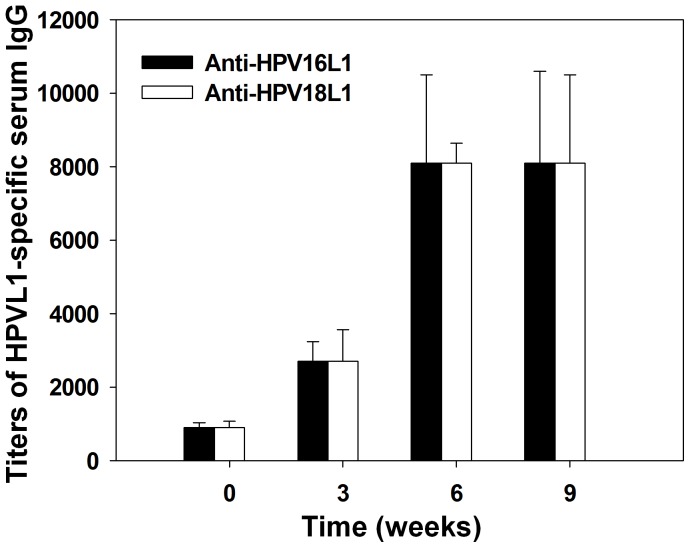
Titer of IgG specific for HPV16L1 and HPV18L1 in porcine sera. Two year-old pigs weighing ∼130 kg were immunized behind the ear by intramuscular injection of AcHERV-HP16/18L1 (3×10^8^ PFU each). Pigs were immunized three times at 3-week intervals. Antigen-specific antibody titers were determined by ELISA.

Finally, we tested antigen-specific serum IgG antibody responses to AcHERV bivalent DNA vaccines in pigs. The intramuscular immunization dose used in these experiments was adjusted to 3.0×10^8^ PFU for both AcHERV-HP16 L1 and AcHERV-HP18 L1 to account for the body weights of the pigs (∼130 kg). Three weeks after the first immunization with the AcHERV bivalent DNA vaccine, anti-HPV16- and anti-HPV18-specific IgG antibodies were detected in serum (Fig. 8), both reaching endpoint titers of 1∶8,100.

## Discussion

In this study, we demonstrated that HERV envelope protein-coated baculoviral vectors could serve as delivery systems for multivalent DNA vaccines in mice and pigs. Co-immunization with AcHERV-based HPV16L1 and HPV18L1 bivalent DNA vaccines provided antigen type-specific humoral serum IgG and vaginal IgA immune responses and induced neutralizing antibodies to an extent comparable to the commercial bivalent VLP vaccine, Cervarix. Notably, the AcHERV-based bivalent DNA vaccines induced cell-mediated immune responses substantially greater than those induced by Cervarix. Moreover, multiple, sequential single immunizations with AcHERV-HP18L1 followed by AcHERV-HP16L1 resulted in the stepwise induction of type-specific IgG antibodies.

We found that AcHERV-based bivalent vaccinations induced antigen-type specific serum IgG (Fig. 3A, 3B) and vaginal IgA antibody responses (Fig. 3C, 3D). Given the multiple HPV type-related pathogenicities, it is essential to develop multivalent rather than monovalent vaccines. Indeed, the currently available VLP vaccines, Cervarix and Gardasil, are bivalent (type 16, 18) and tetravalent (type 6, 11, 16, 18), respectively [22], [23]. Although the current study demonstrated that AcHERV vectors are effective for the delivery of two discrete DNA vaccines, the results obtained indicate the feasibility of applying these AcHERV vectors as a delivery system for multiple DNA vaccines.

The induction of neutralizing antibody is crucial for the prevention of genital tract infection by HPV. Like the currently marketed Cervarix, the AcHERV-based bivalent DNA vaccine induced neutralizing antibody (Fig. 4) to levels that almost completely blocked subsequent challenges with HPV type-specific pseudoviruses (Fig. 5). Given the species restriction of HPV, many HPV vaccine studies have evaluated the immunogenicity by analyzing neutralizing antibodies. In this study we used pseudovirus expressing luciferase to test the cross-protection of immunized mice against other HPV types (Fig. 5). Recently, Christensen’s group developed a challenge model using chimeric HPV capsid/cottontail rabbit papillomavirus genome particles for the direct testing of HPV vaccines in rabbits. In the previous studies, rabbit models have been used for evaluation of chimeric virus-like particle vaccines [24], and N-terminal HPV16 L2 polypeptides [25]. In future study, the cross-protection capability of the AcHERV-based DNA vaccines needs to be further evaluated using the rabbit models.

The fact that AcHERV-based bivalent vaccinations afforded complete protection against challenges with HPV pseudoviruses, a result comparable to that of commercial VLP vaccines, could reflect the effective DNA delivery functions of AcHERV vectors. In our previous study, we reported that AcHERV-HP16L1 notably increased the delivery of HPV16L1 DNA into NIH3T3 human cells lines compared to a baculovirus vector carrying HPV16L1 without HERV *env* [12]. Until now, various papillomavirus L1-based DNA vaccines have been studied in animal models such as rabbits [26]–[29], and beagle dogs [30]. Although DNA vaccines have several advantages over subunit vaccines, one of their major drawbacks is their limited intracellular delivery efficiencies [4]. By coating the surface of a non-replicating baculoviral vector with HERV envelope protein, we were able to increase the intracellular delivery efficiency of DNA vaccines.

Unlike Cervarix, which mainly produced humoral immune responses, inducing only a minimal cell-mediated immune response, AcHERV-based bivalent immunization induced antigen-specific, cell-mediated immune responses (Fig. 6). This inability of current VLP vaccines to induce a cell-mediated immune response limits the suitable target population to adolescent girls with no pre-exposure to HPV infection [31]. To enhance the cell-mediated immune responses of currently available VLP vaccines, researchers have employed approaches to incorporate immune stimulators and block the effect of interleukin-10, which prevents the induction of a cell-mediated immune response [32]. Although efforts have been made to generate a vaccine that can induce cell-mediated immune responses, this goal has been difficult to achieve without using a live attenuated vaccine [33]. In this respect, the induction of both cell-mediated and humoral immune responses by AcHERV-based bivalent DNA vaccines supports the feasibility of developing AcHERV systems for preventive and therapeutic multivalent DNA vaccines, thus expanding the population of those who could benefit from the vaccinations to include individuals who have been previously infected with HPV.

Importantly, we observed that the AcHERV vectors retained effective DNA vaccine delivery functions after multiple injections. To date, several viral vectors have been developed as DNA vaccine delivery systems [34]. One of the limitations of viral vectors for DNA vaccine or gene therapy applications is their induction of antibodies against the viral vector itself or the existence of pre-existing antibodies against the viral vectors, which nullify the effect of the viral vectors after a few doses [34], [35]. To address the feasibility of multiple dosing, we tested the immune responses to separate antigens by sequential immunization of mice with AcHERV-HP18L1 and AcHERV-HP16L1 (Fig. 7). The step-wise induction of anti-HPV18L1 antibody followed by anti-HPV16L1 antibody resulting from this immunization protocol suggests that an AcHERV-based vaccine can function as a multiuse delivery system. The mechanisms that allow repeated dosing of AcHERV vector systems need to be demonstrated at the molecular levels and will require further investigation. However, we speculate that the surface coating of HERV envelope protein on baculovirus nanoparticles prevents elimination by preexisting or induced antibodies and is thus the primary contributor to the persistent in vivo delivery in mice. The clinical implication of the feasibility of repeated dosing is that, once immunized with AcHERV-HPV DNA vaccines, a population could also be immunized in the future with AcHERV vectors carrying other DNA vaccines.

Further development of new vaccine systems requires demonstration of proof-of-concept in large, non-rodent animals in addition to rodent species. The demonstrated induction of humoral immune responses in pigs (Fig. 8) indicates that AcHERV-based bivalent vaccine delivery systems may be effective in large animals. One concern in using pigs as an animal model is that AcHERV vectors are coated with HERV envelope protein, not with protein encoded by porcine endogenous retrovirus *env*. Given the species specificity of endogenous retrovirus, the use of primates in a future study might reveal greater efficacy of the AcHERV-based vectors, possibly reflecting enhanced activation of HERV envelope protein-mediated endocytosis by primate cells. The induction of a humoral immune response in pigs suggests that AcHERV vector-based DNA vaccine delivery systems could be applied against contagious porcine diseases. Moreover, the significant induction of humoral immune responses in pigs weighing 130 kg provides strong evidence that AcHERV systems could prove effective in other large animals, including humans, in addition to laboratory animals. Moreover, we currently construct AcHERV vectors expressing HPV16L1 and 18L1 fusion proteins. It needs to be tested whether the sequential orders of the fusion proteins affect the expression levels and immunogenicity, and whether the AcHERV vectors expressing HPV16L1 and 18L1 fusion proteins provide the immunogenicity comparable to the co-immunization of two separate AcHERV vaccines expressing HPV16L1 and HPV18L1, respectively.

In conclusion, the humoral and cell-mediated immune responses that follow immunization with AcHERV vector-based bivalent DNA vaccines suggest that AcHERV vector systems can be developed as preventive and therapeutic multivalent DNA vaccine delivery systems. Moreover, the persistent immunization efficacy after repeated dosings indicates the future clinical applications of AcHERV vectors as a platform for various DNA vaccines for the same populations.
